# Bone Grafting in Gunshot Fractures of the Jaw

**Published:** 1919

**Authors:** 


**Bone Grafting in Gunshot Fractures of the Jaw.** By Captain WILLIAM BILLINGTON, Dr. ARTHUR H. PARROTT and Dr. HAROLD BOUND, *British Medical Journal*, December 21, 1918.

A report is here made of experiences encountered and results secured in the treatment of injuries of the jaw at the First Southern General Hospital at Birmingham. These patients suffered from compound fracture of the mandible. Successful treatment involved osseous union, functional occlusion, and avoidance of disfigurement. These were accomplished by mechanical technic operated under aseptic conditions. It is essential that firm osseous union be obtained, as otherwise the power of mastication is destroyed. Therefore when the gap was more than half an inch wide a bone graft was used to bridge the cavity.

Two other classes of cases met were those having over-riding or malposition of fragments, and those in which mobility of fragments from muscular action could not be prevented by mechanical means. In these, scar tissue was dissected out, the deformity rectified by division of muscular attachments, and the fragments put in place by means of a plate. If a gap remained, a bone-graft was used.

Bone grafting presented many difficulties, but deformities resulting from allowing fragments to approximate were so great and so disfiguring that surgeons persisted until the present technic was evolved.

Preliminary preparation is prolonged and lasts for months, as the fracture is always complicated by sepsis and often by injury to many soft tissues. X-ray should be made as soon as possible, the patient anesthetized, the wound explored, and foreign bodies removed, together with loose teeth and small fragments of bone. Larger pieces of bone are left in the hope that they will live. It is often possible to do some rough plastic work at this time, putting fragments in place and drawing soft tissues together. Efficient drainage must be provided, and time allowed for healing. Sepsis may be persistent and more than one operation may be necessary. Careful attention must be given to the proper feeding of the patient and care of his general health. Dental splints are very useful in preserving the position of the fragments. Dribbling of saliva must be stopped by plastic work. Soaking with saliva greatly increases the risk of sepsis.

Six weeks after complete healing, the operation of bone-grafting may be done. First, all dental fixation splints must be removed from the mouth. Everything which hinders prompt "healing in"

of graft must be discarded. At operation a skilled anesthetist is most essential.

A curved incision is made in the neck, beginning one inch behind the extremity of the posterior fragment and ending one inch in front of the anterior fragment. This incision begins and ends a half inch above the line of the jaw, and in the neck runs one inch below it, so as to insure a satisfactory flap. Care must be taken not to go into the mouth. The incision is deepened and the tissues raised for an inch and turned up in the flap. Ends of fragments are carefully bevelled away exposing the raw bone. A graft from the iliac crest of the same side having been selected as the best graft material, the graft is prepared in correct contour and fitted into the gap. When first taken out the graft should be two inches longer than the gap to be filled, and is bevelled down to the desired length. If curved bone is desired, that is taken between the superior and inferior spines. No attempt is made to fix these grafts by plates or screws, but they are kept in place by sewing the soft tissues closely over the graft and ends of the fragments with hardened catgut, thus improving nutrition of the graft and diminishing the risk of sepsis. The skin is closed with interrupted stitches, no drainage being employed. No fixation splints are used until healing has taken place, and the wound has been converted into a simple one. Two to four months are required for osseous union, and it is better to allow six months to pass before any dental correction is attempted.

